# Border‐Associated Macrophages: From Embryogenesis to Immune Regulation

**DOI:** 10.1111/cns.70105

**Published:** 2024-11-04

**Authors:** Tiantong Zhan, Sixuan Tian, Sheng Chen

**Affiliations:** ^1^ Department of Neurosurgery, School of Medicine, The Second Affiliated Hospital Zhejiang University Hangzhou China; ^2^ Key Laboratory of Precise Treatment and Clinical Translational Research of Neurological Diseases Hangzhou Zhejiang China

**Keywords:** border‐associated macrophages, central nervous system, embryogenesis, ontogeny, replenishment

## Abstract

Border‐associated macrophages (BAMs) play a pivotal role in maintaining brain homeostasis and responding to pathological conditions. Understanding their origins, characteristics, and roles in both healthy and diseased brains is crucial for advancing our knowledge of neuroinflammatory and neurodegenerative diseases. This review addresses the ontogeny, replenishment, microenvironmental regulation, and transcriptomic heterogeneity of BAMs, highlighting recent advancements in lineage tracing and fate‐mapping studies. Furthermore, we examine the roles of BAMs in maintaining brain homeostasis, immune surveillance, and responses to injury and neurodegenerative diseases. Further research is crucial to clarify the dynamic interplay between BAMs and the brain's microenvironment in health and disease. This effort will not only resolve existing controversies but also reveal new therapeutic targets for neuroinflammatory and neurodegenerative disorders, pushing the boundaries of neuroscience.

## Graphical abstract

Border‐associated macrophages (BAMs) are integral to the maintenance of brain homeostasis and the immune response to pathological conditions. This review addresses the ontogeny, replenishment, and microenvironmental regulation and transcriptomic heterogeneity of BAMs. Additionally, the roles of BAMs in sustaining brain homeostasis, conducting immune surveillance, and mediating responses to injury and neurodegenerative processes are critically examined.

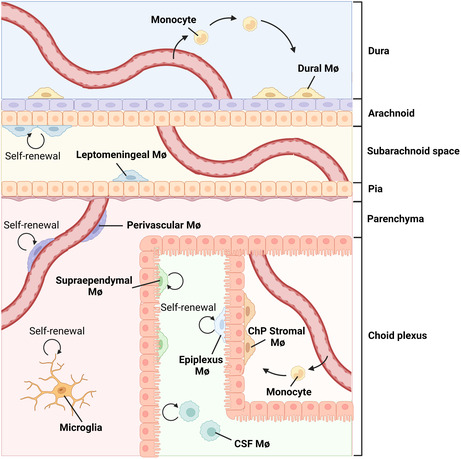

## 1 Introduction

A quarter‐century ago, the brain was believed to operate independently of the immune system. This belief was primarily based on the observation that implanted brain tissues exhibited significantly less immune rejection compared to tissues from other parts of the body [1]. In addition, the highly selective blood–brain barrier (BBB) contributes to the perception of the brain as an “immune‐privileged” organ [2, 3]. This view, however, was challenged in the late 20th century, particularly with the findings highlighting the role of the immune system in autoimmune inflammatory disorders (e.g., multiple sclerosis [MS]) and neurodevelopmental conditions (e.g., autism spectrum disorder [ASD]) [4, 5, 6].

Research has since underscored a positive interaction between the brain and the immune system [7]. Recent studies have further confirmed the critical role of the borders of central nervous system (CNS), including the meninges, perivascular spaces (PVS), and choroid plexus (ChP), in immune surveillance [8, 9, 10]. Comparing to the brain parenchyma, CNS borders serve as the first responders, facing peripheral challenges without disrupting the neuronal network. Additionally, they are in close contact with cerebrospinal fluid (CSF), regulating metabolic waste and other substances. Furthermore, the identification of lymphatic drainage in the CNS borders underscores their importance in maintaining immune homeostasis [11, 12].

Therefore, unlike the brain parenchyma, CNS borders contain a wide repertoire of immune cells in steady‐state conditions [13]. These cells are heterogeneously distributed across different CNS interfaces, performing tissue‐specific functions essential for maintaining CNS integrity [14]. Border‐associated macrophages (BAMs), also known as CNS‐associated macrophages (CAMs), are found in CNS borders [15]. Recent cell‐fate mapping and multimodal high‐throughput and high‐dimensional single‐cell technologies have pointed to a significant heterogeneity of BAMs within the CNS interfaces [15, 16, 17, 18, 19]. Although BAMs and microglia are both myeloid cells and share similarities in morphology, the precise ontogeny, replenishment, transcriptome, and tissue‐specific function of BAMs are still not fully understood. Given the essential roles that BAMs play in neuroinflammation and immunosurveillance, a deeper understanding of these cells is crucial. In this review, we systematically introduce the origin, development, and location of each matured subpopulation of BAMs, providing evidence for their roles in the CNS.

## 2 Comprehensive Insights Into the Origin and Development of BAMs

Over the past few years, several reports on single‐cell analyses of BAMs have unveiled their heterogeneity and diversity [16, 17, 18, 20, 21]. BAMs consist of three main subpopulations: meningeal macrophages (mMs) located in the meninges, perivascular macrophages (pvMs) found in the PVS, and choroid plexus macrophages (cpMs) situated in the ChP. mMs are further divided into dural meningeal macrophages (dmMs) and leptomeningeal macrophages (lmMs). cpMs mainly include epiplexus cells and ChP stromal macrophages. Each subpopulation exhibits unique molecular signatures and specialized functions in immune surveillance and CNS homeostasis maintenance. Each BAM subpopulation originates from CD206^+^ erythromyeloid progenitors (EMPs), whereas dmMs and ChP stromal macrophages are partially replaced by monocytes and retain these characteristics into adulthood [15, 18]. Under the influence of niche factors, BAMs gradually segregate from another type of CNS resident macrophage, microglia, developing unique molecular signatures and specialized functions in immune surveillance and CNS homeostasis maintainance [19].

Macrophages, which are present in most organs, primarily function as tissue sentinels and scavengers by phagocytosing cellular debris and presenting antigens. In addition, macrophages engage in more complex, tissue‐specific roles [22]. Recent understanding suggests that these specialized functions are linked to their ontogeny [23]. During development, organs are colonized by embryonic macrophages derived from precursors in the yolk sac or fetal liver, which can self‐sustain throughout life, in contrast to other subpopulations of macrophages in many tissues that require constant replenishment from bone marrow‐derived monocytes [24, 25, 26, 27, 28]. Another factor influencing macrophage diversity across organs is the distinct molecular programs triggered by the microenvironmental cues in their resident tissues [29, 30, 31]. Therefore, by studying the ontogeny, replenishment, and microenvironmental cues of BAMs, we can gain a comprehensive understanding of this newly discovered cell population.

### 2.1 BAMs Arise From Early Precursors With Mixed Ontogeny

Initially, the embryonic origin of BAMs has been misidentified. Studies transplanting GFP‐transfected bone marrow cells into irradiated rodents revealed that these cells could migrate into the brain and differentiate into BAMs, suggesting a bone marrow origin of these macrophages [32, 33]. However, irradiation could disrupt the BBB and induce chemokine expression, facilitating the influx of bone marrow‐derived cells [34]. Recent research has established that BAM subsets exhibit mixed ontogeny and postnatal development influenced by their border regions [15, 16, 17, 18, 19].

**FIGURE 1:**
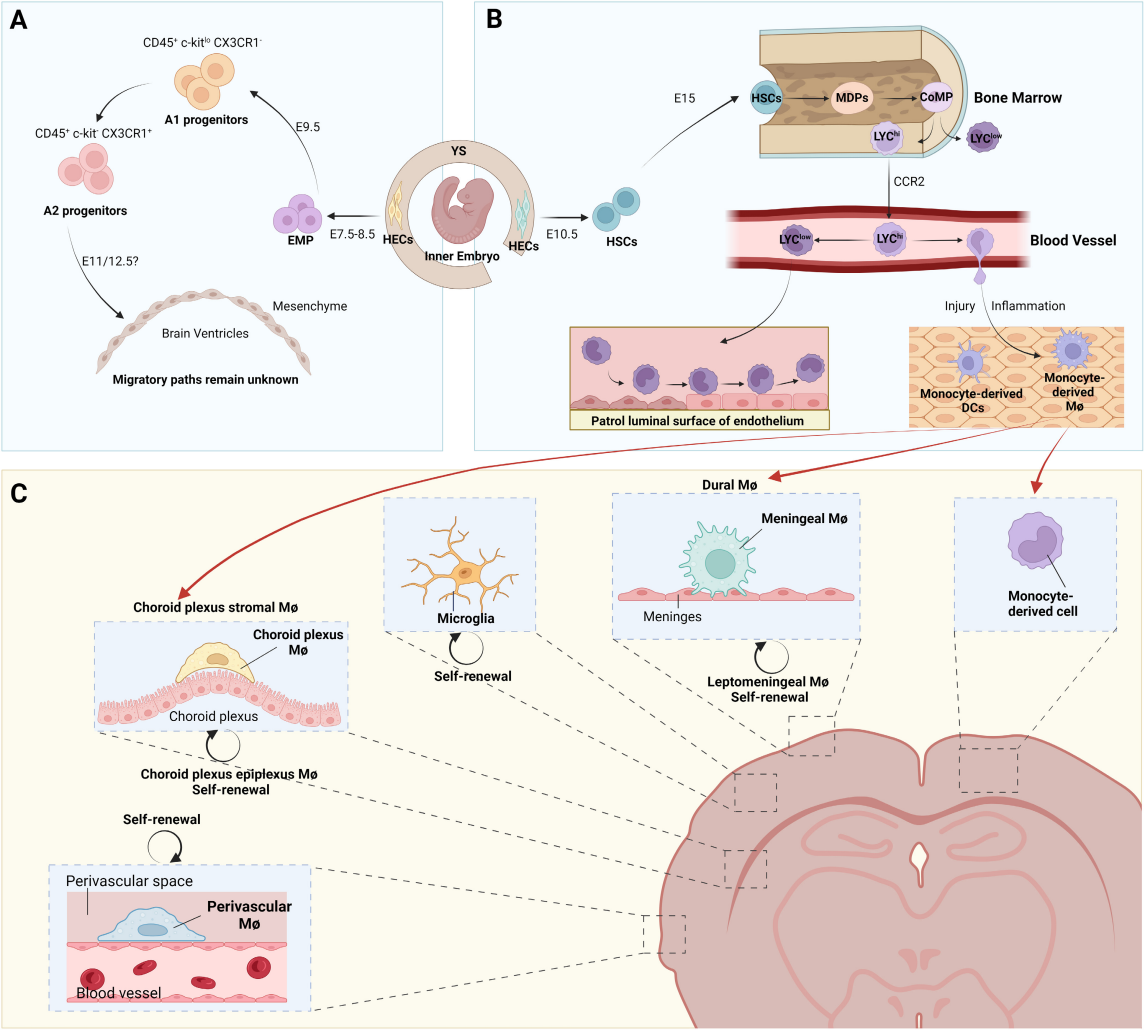
Ontogeny and replenishment of border‐associated macrophages. (A) The emergence of CNS macrophages. The hemogenic endothelial cells (HECs) in the vascular wall of the developing yolk sac (YS) undergo endothelial‐to‐hematopoietic transition (EHT), resulting in the emergence of erythro‐myeloid progenitors (EMP) at E7.5 to E8.5. Around E9.5, EMPs transition from an intermediate immature CD45^+^ c‐kit^lo^ CX3CR1^−^ A1 progenitors, into matured CD45^+^ c‐kit^−^ CX3CR1^+^ A2 pre‐macrophage progenitors, and ultimately differentiate into tissue‐resident macrophages within the central nervous system (CNS). The migration routes of BAMs progenitors remain disputed. (B) Monocyte subsets. At E10.5, hematopoietic stem cells (HSCs) emerge from the hemogenic endothelium located in the aorto‐gonado‐mesonephros (AGM) region, the vitelline, and umbilical arteries. Until E15, they colonize the embryo's bone marrow, and establish it as the primary site of hematopoiesis after birth. They give rise to macrophage and dendritic cell (DC) progenitors (MDPs), differentiating into common monocyte progenitor (cMoP), which give rise to LY6C^hi^ monocytes. They also serve as an intermediate for LY6C^lo^ monocytes generation. Depending on CC‐chemokine receptor 2 (CCR2) for release from the bone marrow, LY6C^hi^ monocytes are swiftly recruited to sites of inflammation and injury, where they extravasate and differentiate into monocyte‐derived macrophages (Mø) and DCs. LY6C^lo^ monocytes, mostly differentiated from LY6C^hi^ monocytes in the circulation, patrol the blood vessel lumen to maintain the integrity of the endothelium. (C) The replenishment of CNS macrophages. As for leptomeningeal Mø, perivascular Mø and choroid plexus epiplexus Mø, once they inhabit their niches, they are proposed to maintain stability through self‐renewal, exhibiting minimal interaction with peripheral myeloid cells, even during autoimmune inflammation, akin to microglia. Conversely, engraftment of peripheral monocytes occurs in the dural mater and choroid plexus stroma after an inflammatory challenge, leading to the replacement of dural Mø and choroid plexus stromal Mø by these monocytes.

In 2016, Goldmann et al. [15] traced the origins of BAMs to the yolk sac using a *Cx3cr1* ^CreER^ *R26* ^YFP^ fate‐mapping mouse model. This finding was further confirmed by Utz et al. Their research indicated that BAMs derive from early EMPs during primitive hematopoiesis, with no contribution from fetal liver monocytes or definitive hematopoiesis (Figure 1A) [19]. However, the origins of different BAM subsets vary. When labeling primitive macrophages at embryonic day (E) 9.0, all Iba1^+^ cells in PVS were labeled at E18.5, whereas only a portion of dmMs and ChP stromal macrophages were labeled [15, 18, 19]. This suggests that pvMs, lmMs, and ChP epiplexus cells retain their embryonic EMP origin into adulthood, whereas macrophages in the dura mater and the ChP stroma are partially replaced by precursors emerging after the appearance of early EMPs and the initial wave of primitive hematopoiesis (Table 1, Figure 1B) [15, 18, 19]. The reasons behind the ontogenetic heterogeneity of BAMs remain unclear. Further research is needed to determine the specific precursors responsible for the partial replacement of dmMs and ChP stromal macrophages, the extent of their replacement, and the mechanisms involved.

**TABLE 1:** Comparative analysis of microglia, border‐associated macrophage (BAMs) and monocytes/ bone marrow‐derived macrophages (BMDMs).

	Microglia	Border‐associated macrophages (BAMs)	Monocytes/bone marrow‐derived macrophages (BMDMs)
Origin	Yolk sac [15]	Yolk sac; partial replacement by monocytes of dmMs and ChP stromal macrophages [15, 18, 19]	Hematopoietic stem cells (HSCs) in bone marrow [68]
Location	Brain parenchyma [15]	Meninges, perivascular spaces, choroid plexus [15]	Blood stream and tissues [134]
Self‐renewal	Yes [135]	Varies by subsets, pvMs, lmMs and ChP epiplexus cells self‐renew [18]	No; replenished from bone marrow [68]
Replacement by monocytes	Rare [135]	Specific subsets, dmMs and ChP stromal macrophages are replenished by monocytes [18, 44]	Yes; recruited to sites of infection/injury [136]
Turnover rate	Low; long‐lived	Varies; pvMs, lmMs and ChP epiplexus cells are long‐lived, dmMs and ChP stromal macrophages have higher turnover rates [18, 44]	High; regularly replenished by bone marrow [137]
Transcriptomic profile	*P2ry12*, *Tmem119*, *Sall1*, *Sall3*, *Hexb*, *Siglech*, *Olfm13* Highly stable [17, 138]	*Mrc1*, *Ms4a7*, *Pf4*, *Tgfbi*, *Lyve1*, *Cd163*, *Siglec1* [17, 138] Heterogeneous, distinct profiles in different regions	*Ly6c*, *Ccr2*, *Fn1*, *Cd44* [17] Dynamic, can rapidly change in response to environment
Surface markers	TMEM119 [139]	CD163, CD169, CD206, CD38, Lyve1 [15, 16, 140, 141] Heterogeneous, distinct profiles in different regions	CD14, CD16, CD11b, CCR2, Ly6C [17]
Mobility	Low mobility [142]	Varies; pvMs and mMs have limited mobility [15, 34, 64, 143, 144]; cpMs present higher mobility responding to injury and inflammation [109]	High mobility, migrate quickly to sites of infection or injury [145]
Morphology	Small cell body with fine, branched processes [146, 147]	Morphology varies with location [15, 64, 141]	Round or oval shaped; larger cell body when differentiated into macrophages [145]

Abbreviations: ChP, choroid plexus; dmMs, dural meningeal macrophages; lmMs, leptomeningeal macrophages; pvMs, perivascular macrophages.

### 2.2 BAMs and Microglia Share a Common Progenitor

Despite both BAMs and microglia originating from EMPs, they still exhibit distinct transcriptomes profiles in the adult CNS [18]. For instance, adult BAMs can be differentiated from microglia by their distinct expression of markers such as Siglec1 (CD169), CD206, Lyve1, CD38, MHCII, CD11c, and CD163 [16]. Conversely, microglia do not express these markers but do express *Tmem119*, *P2ry12*, *Sall3*, and *Sall1* (Table 1) [30, 35, 36, 37]. This observation has led to discussions about whether they share a common progenitor and acquire their differences within the CNS niches, or if the heterogeneity is already present in the yolk sac progenitor cells.

Utz et al. [19] identified two distinct primitive macrophage populations, CD206^−^ and CD206^+^, diverging in the yolk sac and brain as early as E10.5. Since CD206 (encoded by *Mrc1*) is predominantly expressed by adult BAMs and absent in homeostatic adult microglia [15, 17, 18], CD206^−^ macrophages can be identified as microglia progenitors and CD206^+^ macrophages as BAM progenitors. This indicates that progenitors for microglia and BAMs are present in the embryonic brain as early as E10.5 [19]. Masuda et al. [21] performed fate‐mapping analysis of the CD206^+^ yolk sac subpopulation by injecting 4OH‐Tamoxifen (TAM) into *Mrc1* ^*CreERT2/CreERT2*^ *R26* ^*tdT/tdT*^ mice. When TAM was administered at E9.0, both microglia and BAMs were marked with tdTomato (tdT) by postnatal day (P) 14. However, when TAM was administered at P14 and P16, only BAMs were labeled with tdT, while microglia were not [21]. This observation indicates that BAMs and microglia originate from a common CD206^+^ yolk sac progenitor. These progenitor cells later colonize different regions of the brain during development, with segregation completing postnatally. Additionally, BAMs gradually differentiate. Some BAMs retain their original progenitor characteristics, resembling microglia in traits and self‐renewal capabilities [15]. Conversely, other BAMs change over time, expressing peripheral monocyte genes and undergoing replacement by monocytes in adulthood [15]. These transformations, driven by the local microenvironment, lead to increased diversity among the cell groups (Table 1).

### 2.3 Local Microenvironment Shapes BAM Characteristics

Researchers have studied the divergence in the differentiation pathways of microglia and BAMs. From E9.5 onward, the progenitors of BAMs and microglia began to occupy distinct niches and gradually developed their unique characteristics [38]. These processes were mediated by local factors, such as cell–cell interactions, metabolites or cytokines, to activate or inhibit transcription factors, resulting in unique transcriptomics, phenotypes, and functions to adapt the local environment [39]. Both BAMs and microglia depend on colony‐stimulating factor 1 receptor (CSF‐1R) signaling [16, 18, 24] and the transcription factors PU.1 and IRF8 [15, 18, 40]. However, recent multi‐parametric single‐cell analyses have identified that only microglia depend on transforming growth factor β (TGF‐β) and SMAD4 (the downstream transcription factor of TGF‐β signaling pathway), whereas BAMs develop and maintain independently of them [19, 41]. Researchers showed that in the absence of these specific transcription factors, the transcriptome and epigenome of microglia become almost indistinguishable from that of BAMs, and thus suggesting the microenvironment crucially controls the specification program of microglia and BAMs [41].

Under the influence of local factors, the morphologies of BAMs and microglia change upon seeding their respective niches: microglia acquire a characteristic ramified appearance, whereas BAMs retain an amoeboid shape throughout development [19]. From E12.5 onward, the molecular signatures of microglia and BAMs diverge further, adopting characteristics similar to their adult profiles [16, 17, 18]. As niche factors continue to influence their development, the initially homogeneous embryonic BAM population become increasingly heterogeneous during maturation. The differences in niche factors received by different subpopulations still require further investigation.

### 2.4 Monocytes Replenishment in Adult BAM Maintenance

The heterogeneity of BAMs is further shaped by contributions from monocytes, a characteristic that persists into adulthood [20, 42, 43]. Adult lmMs, pvMs, and ChP epiplexus macrophages are proposed to maintain stability through self‐renewal and exhibit minimal exchange with peripheral myeloid cells, even under perturbations [15]. On the contrary, the engraftment of peripheral monocytes occurs in the dural mater and ChP stroma during homeostasis or following an inflammatory challenge (Table 1, Figure 1C) [18, 44]. dmMs and ChP stromal macrophages are reminiscent of Ly6C^hi^ monocytes and show high levels of *Ly6c2*, *Ccr2*, *Fn1*, and *Cd44* [17]. This suggests that tissue accessibility significantly influences brain macrophage replenishment: more permeable regions, such as the dura mater and ChP stroma, are prone to bone marrow replacement, unlike the brain's more protected regions. Additionally, certain perturbations, such as physical damage to the vasculature and CNS barriers (e.g., stroke) and neuroinflammatory diseases (e.g., infections), are more likely to affect these areas, facilitating the massive infiltration of peripheral immune cells [45].

## 3 Meningeal Macrophages

The meninges, composed of the dura mater (pachymeninx) and the leptomeninges (pia and arachnoid maters), serve as the first line of defense against microbial threats and as a gateway for immune cell infiltration [46, 47]. To maintain homeostasis, the meninges host diverse immune cells, including mMs, which are a type of BAMs [15]. These mMs can be classified into two subpopulations based on their anatomical locations: dmMs and lmMs [18].

As the meninges undergo ontogeny, niches gradually develop and become available for the embryogenesis and maturation of mMs. In mice, Iba1^+^ mMs are detected in the primary meninx as early as E10.5 when the first fibroblasts form a primary Col IV^+^ meninges [21]. During development, the expression of BAM core signature genes (e.g., *Pf4*, *Lyve1*, and *Mrc1*) in mMs changes dynamically, peaking at the early postnatal stage [21]. Specifically, embryonic and postnatal mMs are predominantly MHCII^−^. However, during childhood, MHCII^+^ mMs begin to appear [18, 19]. Recently, Brioschi et al. [41] indicated that CD38^+^ MHC II^−^ BAMs in the brain are predominantly of embryonic EMP origin, whereas CD38^−^ MHC II^+^ BAMs are derived from monocytes. This phenomenon may indirectly confirm that mMs are partially replaced by monocytes that emerge after the appearance of early EMPs.

Adult mMs display active processes akin to microglia, constantly surveying their surroundings [9, 44]. Homeostatic mMs express genes such as *Mrc1*, *Pf4*, *Cbr2*, *Ms4a7*, *Stab1*, *Fcrls*, *Cd163*, and *Siglec1* [17]. They also express common macrophage markers (CD11b, F4/80, and CD64), along with costimulatory markers (CD80), adhesion molecules (intercellular adhesion molecule 1 [ICAM 1]), and activated macrophages (CD206) [9, 44]. Besides, adult mMs can be further subdivided into two subpopulations based on major histocompatibility complex (MHC) II expression [18, 19].

Due to their distinct anatomical locations, dmMs and lmMs encounter different immunological challenges. To adapt, these subpopulations develop unique morphological characteristics and transcriptomic profiles, enabling them to perform specific functions [48].

### 3.1 Dural Meningeal Macrophages

#### 3.1.1 Anatomy and Macrophage Distribution of the Dura Mater

**FIGURE 2:**
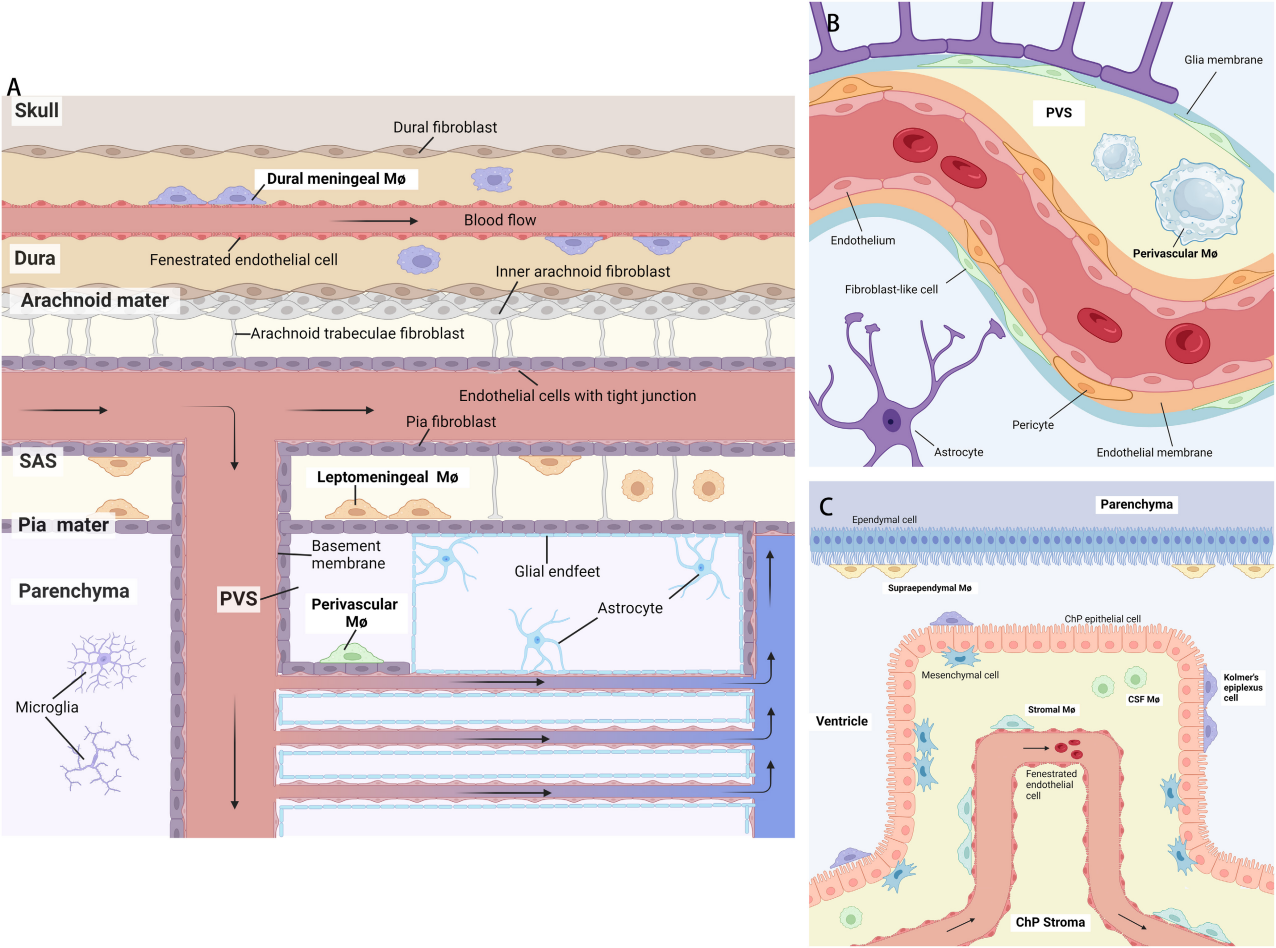
Anatomical structure of border‐associated macrophages niches. (A) Localization of border‐associated macrophages in the meninges. Dural capillary endothelial cells lack tight junctions and thus allow leakage of peripheral molecules into the dura mater. In the dural sinus, dural meningeal Mø mainly line the blood vessels or the lymphatics (not illustrated), but there are also interstitial dural meningeal Mø. The vascular endothelial cells of subarachnoid space (SAS) and arachnoid barrier cells express tight junctions, establishing the meningeal blood–CSF barrier. Leptomeningeal Mø inhabits the CSF‐filled SAS, in close proximity to the vasculature. Blood vessels that submerge into the CNS parenchyma carry a layer of pial fibroblast with them. Vascular endothelial cells express tight junctions and establish the blood–brain barrier (BBB). The glia limitans, which consist of the parenchymal basement and astrocytic endfeet, constitute with the vascular smooth muscle cells, forming the perivascular spaces (PVS), the niche for perivascular Mø. Microglia are the only macrophages found in the CNS parenchyma. (B) Localization of border‐associated macrophages in perivascular spaces (PVS). The vascular endothelial cells of parenchymal vessels are non‐fenestrated, constituting a basement membrane. Along arterioles and veins, the distance between the vascular and glial basement membranes is larger, forming the PVS. Vascular smooth muscle cells, fibroblasts and pericytes can be found in the PVS, constituting the niche for perivascular Mø. (C) Localization of border‐associated macrophages in the choroid plexus (ChP). Ependymal cells seal the brain ventricles, connecting with the epithelial cells which form the surface of ChP. Beneath the epithelial layers, mesenchymal cells are situated, providing structural support. The stroma core of ChP consists of fenestrated capillaries without tight junctions, allowing permissiveness of water and small molecules. Epiplexus cells (or Kolmer's epiplexus cells) seed on the apical surfaces of the ChP epithelium. Between the epithelial and endothelial layers, stromal ChP macrophages are observed, generally within the stromal tissue alongside mesenchymal cells. Supraependymal macrophages (attached to ependymal cells) and free‐floating CSF macrophages are observed as well.

The characteristics and functions of dmMs are shaped by the local microenvironment of the dura mater. This highly vascularized tissue facilitates extensive substance exchange, presenting significant immunological challenges [49, 50]. Due to the absence of tight junctions and the presence of fenestrated capillaries, the dura mater cannot establish a BBB, allowing rapid exchange of blood‐derived molecules and cells [49, 51]. Additionally, the dura mater contains a network of lymphatic vessels that drain CNS‐derived macromolecules to the deep cervical lymph nodes [11]. This structure allows dura mater access to external pathogens and metabolic waste, leading to an abundant and diverse immune cell population in the region [47, 52]. Consequently, the dural sinus, adjacent to blood vessels penetrating the dura, harbors dense populations of immune cells, including dmMs (Table 2, Figure 2A) [8, 12, 53]. Living in this environment, dmMs are predominantly MHCII^+^ and actively serve as sentinels of the immune defense, gaining the ability to present antigens [16]. Their morphology and transcriptomic profiles have also changed to adapt to the local microenvironment.

**TABLE 2:** Cellular composition and anatomical locations of border‐associated macrophage niches.

	Dura	Leptomeninges			PVS	ChP			
Vascular network	Arteries, veins, capillaries, venous sinuses [50]	Arteries, veins, without capillary network [49]			Arteries, veins [61]	Capillaries (stroma core)			
Endothelial cell‐to‐cell junctions	Lack of tight junctions [51]	Tight junctions [49]			Tight junctions [61]	Lack of tight junctions [50, 51, 101]			
Epithelial cells	No epithelial layer [45]	Arachnoid mater		Pia mater	Glia basement membrane & vascular basement membrane [61]	Brain ventricles		ChP surface epithelium	
		Arachnoid barrier cells	Fibroblasts	Leptomeningeal cells		Ependymal cells [101]	ChP epithelial cells, mesenchymal cells [101]		
Epithelial cell‐to‐cell junctions		Tight junctions [49]	Desmosomes [49]	Desmosomes [49]		Gap junctions [101]		Tight junctions [101]	
Barriers	Do not establish BBB [46]	Meningeal blood–CSF barrier			Glia limitans [61]	Circumventricular organ barrier [101]		Blood–CSF barrier [101]	
BAM subtype	dmMs [15, 16, 17]	lmMs [15, 16, 17]			pvMs [15, 16, 17]	Supraependymal Mø [104]	CSF Mø [104]	Epiplexus cells [104]	ChP stromal Mø [104]
BAM location	Dural sinuses and non‐sinusoidal spaces [15, 16, 17]	CSF‐filled SAS, in close proximity to the vasculature [15, 16, 17]			PVS [15, 16, 17]	Attached to ependymal cells [104]	Free‐floating [104]	On the apical epithelium [104]	Between the epithelial and endothelial layers [104]

Abbreviations: BAMs, border‐associated macrophages; BBB, blood–brain barrier; ChP, choroid plexus; CSF, cerebral spinal fluid; dmMs, dural meningeal macrophages; lmMs, leptomeningeal macrophages; Mø, macrophag; pvMs, perivascular macrophages; PVS, perivascular spaces.

#### 3.1.2 Sentinel Roles of dmMs in Immune Defense

##### 3.1.2.1 Dura Mater Maintains dmMs in a Surveillance Phenotype

The dura mater's vasculature facilitates the coordinated recruitment, extravasation, and retention of peripheral immune cells, particularly T cells and B cells, thereby promoting their interactions with dmMs [8]. This process operates through two main mechanisms: physical and signaling interactions (e.g., adhesion molecule‐integrin binding and chemokine–chemokine receptor signaling), and local development and retention of immune cells. Single‐cell RNA sequencing data have unveiled that the dural vasculature exhibit significant expression of adhesion molecules including *Vcam1*, *Icam1*, and *Selp* [8]. Moreover, fibroblast‐like cells in the dura produce stromal cell‐derived factors that promote B cell development and T cell retention. This leads to an enrichment of immune cells and establishes these regional hubs as crucial points for the interaction between peripheral and CNS immune systems, enabling dmMs to act as antigen presenting cells (APCs) [8, 54].

Min et al. [55] indicated that dural mural cells, such as pericytes and smooth muscle cells, regulate the immune response between dmMs and T cells. Under steady‐state conditions, mural cells inhibit the infiltration of exogenous T cells into the dura mater. To prevent abnormal activation of dmMs, mural cells suppress dmM‐mediated antigen presentation to T cells by transferring cytoplasmic components, such as RNA granules, to reprogram their transcriptomes [55]. However, under perturbations such as experimental autoimmune encephalomyelitis (EAE), the coverage of mural cells along dural blood vessels is reduced [55]. This reduction leads to the loss of the protective function of mural cells over the dura mater, resulting in decreased inhibition of dmMs and increased T cell enrichment, thereby enhancing the antigen‐presenting function of dmMs [55].

##### 3.1.2.2 Disease‐Specific Antigen Processing by dmMs

During inflammation, MHCII^+^ dmMs capture and present CNS‐derived antigens to patrolling T cells, facilitating immune surveillance and tissue retention [8]. Their antigen‐presenting capabilities vary across different diseases. In viral meningitis mouse model infected with lymphocytic choriomeningitis virus (LCMV), MHC II^+^ dmMs become activated in the dura mater, acquiring viral antigens and leading to their significant depletion [44]. However, some researchers suggested that MHC‐II‐mediated antigen presentation may not be necessary for dmM antiviral functions. Instead, they proposed that dmMs utilize the interferon (IFN) response through the Stat1 pathway to amplify antiviral effects and suppress further viral infection [56].

However, in autoimmune conditions like EAE, the number of MHCII^+^ dmMs remains constant. Although T cells accumulate massively in the dura mater, their activation is limited [8, 17]. dmMs are considered to be unable to fully activate T cells and do not present sufficient autoantigens in vivo, whereas leptomeningeal APCs load higher levels of autoantigens [57]. This may be because autoantigens do not reach the dura in sufficient quantities, resulting in a lower degree of relevant autoantigen presentation by dmMs [57].

##### 3.1.2.3 dmMs Require Monocyte Replenishment

dmMs show high levels of *Ly6c2*, *Ccr2*, *Fn1*, and *Cd44*, which confirmed as being highly expressed by peripheral monocytes [17]. The highly fenestrated vasculature and tissue structure of the dura mater also facilitate the infiltration of peripheral monocytes, compensating for the depletion of dmMs caused by metabolism or disease. Following the depletion of dmMs, the meningeal niche is replenished within a month, driven by robust monocyte recruitment from the meningeal blood vessels [44]. Furthermore, Cugurra et al. discovered bone marrow niches in the skull and vertebrae adjacent to the dura mater. These niches serve as myeloid reservoirs, providing monocytes that directly seed the dura mater and differentiate into dmMs [58]. This timely replenishment allows dmMs to continue participating in immune responses.

### 3.2 Leptomeningeal Macrophages

#### 3.2.1 Anatomy and Macrophage Distribution of the Dura Mater

Compared to the dura mater, which has high permeability and a diverse population of immune cells, the leptomeninges are less permeable and host fewer types of immune cells. The arachnoid mater acts as a vascular barrier, with cells connected by tight junctions to form the meningeal blood–CSF barrier. Its inner layer consists of densely packed fibroblasts connected by desmosomes [49]. The pia mater, located close to the parenchyma, is composed of a single layer of leptomeningeal cells with less frequent gap junctions by desmosomes, making it permissive to limited immune cells [50, 51]. The subarachnoid space (SAS), enclosed by the leptomeninges and filled with CSF, contains arteries and veins but lacks a capillary network [49]. The endothelial cells of SAS vessels express tight junctions without fenestrations. Therefore, the immune cells in the SAS are less diverse and are mostly lmMs under homeostatic conditions [15, 16, 17, 18]. These lmMs are in close proximity to the vasculature and may interact with it, although their interconnections are not fully understood (Table 2, Figure 2A).

#### 3.2.2 MHCII ^+^ lmMs Present Autoantigens With Higher Efficiency

Despite the structure of the SAS limiting contact between lmMs and peripheral immune cells, research indicates that MHCII^+^ lmMs present autoantigens more efficiently than MHCII^+^ dmMs. This leads to higher levels of T cell infiltration and activation in the leptomeninges in both experimental models and patients with MS [57]. Several factors contribute to this phenomenon. The leptomeningeal endothelial cells and lmMs are more adhesive to T cell infiltration and attachment, expressing higher levels of integrin ligands and chemokines than those in the dura [57]. Even when T cells detach from the leptomeninges into the CSF, they can reattach to the leptomeninges, re‐triggering local immune responses with lmMs [59]. In addition, compared to the dura, more autoantigens can reach the leptomeninges [57]. These factors collectively enable lmMs to present autoantigens to the infiltrating T cells with higher efficiency, thereby enhancing their role in immune surveillance.

#### 3.2.3 MHCII ^−^ lmMs Regulate CSF Flow Dynamics

LYVE‐1 is the receptor of hyaluronan. Lim et al. reported that in mouse aorta, Lyve1^+^ resident macrophages could modulate collagen expression in vascular smooth muscle cells (VSMCs) by matrix metalloproteinase (MMP)‐9‐dependent proteolysis through engagement of LYVE‐1 with the hyaluronan extracellular matrix (ECM), regulating normal physiological arterial function [60]. Although dmMs are predominantly Lyve1^−^, lmMs and pvMs both express LYVE‐l and are in close proximity to the vasculature, suggesting their potential role in maintaining appropriate arterial tone [16].

Drieu et al. found that MHCII^−^ lmMs and pvMs can regulate CSF flow under both homeostatic and pathological conditions [10]. After depletion of these macrophages, the activity of MMPs decreases, and ECM proteins such as collagen type IV and laminin accumulate in proximity to the blood vessels. This accumulation interferes with ECM degradation and remodeling, potentially affecting arterial stiffness and vasodilatory response. This indicates that MHCII^−^ lmMs and pvMs regulate arterial motion, a major driver of CSF flow dynamics, thereby maintaining CSF homeostasis [10].

## 4 Perivascular Macrophages

### 4.1 Overviews of pvMs

#### 4.1.1 Anatomy and Macrophage Distribution of the PVS

In adult mice, astrocytes extend their endfeet to form a glia parenchymal basement membrane beneath the pia mater. Vessels sprouting from the SAS penetrate into the parenchyma, forming a non‐fenestrated vascular basement membrane due to tight junctions between endothelial cells [61]. Along arterioles and veins, the glial and vascular membranes fuse and form the PVS, also known as the Virchow‐Robin space [62]. Within the PVS, a subset of BAMs resides, known as pvMs [15]. Mature pvMs predominantly localize around arteries and arterioles that express alpha smooth muscle actin (aSMA) and SRY‐box transcription factor 17 (SOX17), but do not express transferrin receptor (TfR) [21]. A small population of these cells can also be observed around non‐muscularized aSMA^+^ TfR^+^ venules in the PVS (Table 2, Figure 2B) [63]. The unique location of pvMs in the PVS around blood vessels shapes their morphology. Unlike the highly ramified microglia, pvMs have a simpler, elongated structure that allows them to surround vessel walls with protrusions extending and retracting along these walls [15, 64].

#### 4.1.2 The Postnatal Shift of pvMs

The emergence of classical pvMs occurs postnatally, concurrent with the establishment of the PVS [21]. Around E9.5, angioblasts begin penetrating the neuroectoderm, and by E10.0, vascular endothelial cells start expressing tight junctions [65, 66]. However, other cellular constituents, such as astrocytes and VSMCs, show a developmental delay compared to endothelial cells. Astrogliogenesis in the cerebral cortex starts around E18.0 and continues beyond P7 [67]. Similarly, arteries or arterioles containing basal lamina or VSMCs are not detected until P3 [21]. By P10, CD206^+^ LYVE1^+^ P2RY12^−^ macrophages appear in the PVS, characterizing typical pvMs [21]. In human, the shift of pvMs also occurs postnatally. During the first 25 weeks of gestation, CD206^+^ pvMs are barely detectable in the fetal brain, but increase significantly after birth [21].

pvMs temporarily reside in a specific niche until the PVS, especially the VSMCs, fully develop. After birth, pvMs establish and distribute themselves in the PVS through local proliferation [21]. Besides, the leptomeninges can also serve as an intermediate niche, allowing the migration of lmMs into the PVS, contributing to pvM development [21]. Additionally, the interaction between VSMCs and pvMs promotes the migration of pvMs, facilitating their integration into the perivascular niche [10].

#### 4.1.3 Transcriptomic Profiles of pvMs

Adult pvMs exhibit typical transcriptomic profiles of BAMs. They show the Lyve1^+^ CD45^hi^ phenotype and express key macrophage markers such as F4/80, CD11b, CD206, Iba‐1, Csf‐1R, and CX3CR1, with gene expression including *Lyve1*, *Mrc1*, *Cd163*, and *Maf* [15, 68, 69]. Recently, a rare subset of pvMs in the brain parenchyma characterized by CX3CR1^−^ Lyve1^+^ F4/80^+^ Iba1^+^ CD206^+^ CD45^lo^ expression has been identified, although the specific functions of this subset remain unclear [69].

### 4.2 pvMs in Immune Surveillance and Homeostasis

The PVS contain CSF, which includes substances that have crossed the BBB from the peripheral circulation [70]. These spaces also facilitate the exchange of CSF with the interstitial fluid (ISF), aiding in the removal of metabolic waste through the glymphatic system [71, 72, 73]. Within the PVS, pvMs play crucial roles as tissue sentinels and scavengers, contributing to immune surveillance by acting as APCs and maintaining homeostasis by phagocytosing cellular debris [74].

The ability of pvMs to function as APCs is evidenced by their expression of MHC molecules [75, 76]. Under physiological conditions, CD4^+^ T cells can be recruited to the PVS either directly from the CSF or by crossing the endothelial barrier from the peripheral bloodstream [77, 78]. pvMs take up and process foreign antigens, presenting them to CD4^+^ T cells through MHC II molecules [79, 80, 81, 82]. Once activated by specific antigens in the PVS, T cells can migrate into the CNS parenchyma via the glia limitans, potentially causing inflammatory tissue damage and clinical symptoms [83]. For instance, in an EAE rodent model, CD4^+^ T cells are discovered to be compartmentalized within the PVS until they recognize their cognate antigen on pvMs before migration [84, 85]. pvMs and dendritic cells can release proinflammatory cytokines such as interleukin (IL)‐23 in the PVS, further stimulating autoreactive T cells locally [86]. Besides, MHC I receptors on pvMs are responsible for promoting CD8^+^ T cell infiltration into the brain during viral infection [87].

The scavenging function of pvMs is attributed to the presence of CD206, a transmembrane glycoprotein involved in the uptake of glycoproteins and carbohydrate‐containing structures, as well as the scavenger receptor CD163 [88, 89]. It has been shown that β‐amyloid (Aβ) is drained along the PVS, and the phagocytosis of Aβ by pvMs is considered protective [90]. A study demonstrated that ablation of pvMs with clodronate liposomes in TgCRND8 mice worsened cerebral amyloid vasculopathy [91]. However, clodronate liposomes are not specific to pvMs and also deplete other macrophages, although they do not affect microglia and astrocytes [92]. Scavenger receptor class B type I (SR‐BI), a high‐density lipoprotein (HDL) receptor expressed in pvMs, regulates amyloid plaque deposition in the cortex and hippocampus [93].

### 4.3 pvMs in Neurovascular Dysfunction and BBB Integrity

pvMs, closely associated with cerebral microvessels, are major contributors to neurovascular dysfunction and play a role in maintaining BBB integrity [74]. One mechanism involves Nox2 in pvMs, which regulates BBB permeability by affecting vascular oxidative stress [94, 95]. During hypertension, circulating angiotensin II (Ang II) engages angiotensin II Type 1 Receptor (AT1R) in cerebral endothelial cells leading to increased transcytosis and tight junction remodeling, which results in increased BBB permeability. This allows the peptide to gain access to the PVS and activate AT1R in pvMs. The resulting increase in Nox2‐dependent vascular oxidative stress, in turn, enhances the endothelial dysfunction and BBB leakage, potentially leading to cognitive impairment [95]. In Alzheimer's disease, elevated Aβ in the PVS activates CD36 in pvMs, triggering a Nox2‐containing nicotinamide adenine dinucleotide phosphate (NADPH) oxidase, leading to cerebrovascular oxidative stress responsible for the cerebrovascular effects of Aβ [94, 96, 97].

pvMs can limit the enhanced permeability of the BBB directly. In the area postrema, where the BBB is less restrictive due to its fenestrated microvasculature, CD163^+^ pvMs form a barrier by phagocytosing blood‐borne macromolecules and serum proteins [98]. However, the exact mechanisms of this process are not yet fully understood. In the peripheral nervous system, pvMs have been observed to regulate the permeability of the blood–nerve barrier, which shares similar functional and morphological characteristics with the BBB but is more permeable [99]. Lund et al. discovered that pvMs in sensory ganglia exhibit a vasculature‐associated transcriptional profile, make close contact with endothelial cells, receive survival signals from endothelial‐associated pericytes, rapidly phagocytose circulating macromolecules, and increase vessel coverage in response to circulating endotoxin, thereby maintaining the integrity of the BBB [100]. The mechanisms identified in this process may offer insights into how CNS pvMs modulate BBB permeability.

## 5 Choroid Plexus Macrophages

### 5.1 Overviews of cpMs

#### 5.1.1 The Anatomical Features and Macrophage Residence of the ChP

The ChP is a critical structure within the brain's ventricles, primarily responsible for producing CSF. This function is facilitated by the stroma core containing fenestrated capillaries, which allow the passage of water and small molecules [101, 102]. The stroma core is surrounded by a monolayer of ciliated cuboidal epithelial cells connected by tight junctions, forming the blood–CSF barrier (BCSFB). This barrier prevents the uncontrolled entry of blood‐derived molecules into the CSF‐filled ventricles [101, 102]. Beneath the epithelial layer are mesenchymal cells, providing structural support and maintaining the integrity of the ChP [101, 102]. The brain ventricles, interconnected and filled with CSF, are lined with ependymal cells, specialized glial cells that facilitate the diffusion of metabolic waste and nutrient exchange through gap junctions [101, 102].

Numerous immune cells reside in the ChP, with cpMs being the most prominent [103]. cpMs can be subcategorized based on their specific locations. Epiplexus cells, or Kolmer's cells, are found on the apical surfaces of the ChP epithelium. Between the epithelium and endothelium, ChP stromal macrophages are generally located within the stroma alongside mesenchymal cells [104]. Other types of intraventricular macrophages, such as supraependymal macrophages (attached to ependymal cells) and free‐floating CSF macrophages, are also present (Table 2, Figure 2C) [104]. The morphological characteristics of cpMs vary significantly, including spherical, bipolar, and stellate shapes [104]. Typically, adult homeostatic cpMs have a smooth, centrally positioned cell body with three to five prominent cytoplasmic extensions [104].

Given the limited research on other types of intraventricular macrophages, this section focuses on epiplexus cells and ChP stromal macrophages.

#### 5.1.2 Development of cpMs

In mice, the initial CSF system forms between E9 and E9.5 following the closure of the neural tube [104]. After closure, the cephalic end of the neural tube dilates, gradually forming different vesicles where ChP tissue emerges sequentially between E11.5 and E13.5 [105]. This tissue rapidly establishes barrier properties and begins secreting CSF, nourishing the developing brain [105]. cpMs are first observed in the developing CSF system at E11.5 [106, 107]. During development, ChP stromal macrophages are gradually replaced by bone marrow–derived cells, likely entering the stroma through diapedesis across the fenestrated choroidal vasculature [18, 104]. In contrast, epiplexus cells persist through self‐renewal of embryo‐generated macrophages [18].

Recent findings suggest that the development and aging regulation of cpMs is subpopulation‐specific and involves interactions with structural cells. The development of epiplexus cells depends on the *Csf1r* enhancer, a signal crucial for macrophage and monocyte differentiation, whereas the development of ChP stromal macrophages is only partially dependent on this enhancer [107]. The mesenchymal and endothelial cells of ChP express *Csf1*, suggesting their role in cpM maturation, which might influence cpMs to varying degrees [103]. Additionally, in aged cpMs, IL‐1β expression is elevated, with its receptor, IL‐1 receptor type 1 (IL‐1R1), also upregulated in the endothelial cells of vessels connecting the ChP and parenchyma. This ligand‐receptor interaction likely promotes the migration and infiltration of aged cpMs through the ChP‐brain barrier, potentially initiating local immune responses in the parenchyma [103].

#### 5.1.3 Transcriptomic Profiles of cpMs

The transcriptomic signatures of cpMs change during development and aging. In adult and aged cpMs, *Cd206* is downregulated, whereas *Cd74*, a chaperone of antigen presentation, is enriched [18, 103]. Additionally, inflammatory signals such as *Il1b* are upregulated in aged cpMs, reflected by *Il1r1* expression in aged ChP epithelial cells [103].

Most adult cpMs share signature genes with adult BAMs and are *Sall1* negative, except for epiplexus cells, which have a microglial transcriptome signature, including *Sall1*, *P2ry12*, and *Slc2a5* [17, 18, 20]. This may suggest that epiplexus cells and microglia share a common origin, and consequently, like microglia, epiplexus cells are not replenished by circulating monocytes [18]. Homeostatic epiplexus cells also exhibit gene expression similar to disease‐associated microglia (DAM), displaying reduced expression of homeostatic microglia genes (e.g., *P2ry12*, *Tmem119*, and *Hexb*) and increased expression of genes linked to phagocytosis and lipid metabolism (e.g., *Lpl*, *Apoe*, *Clec7a*, and *Cst7*) [18, 104, 108].

cpMs in distinct spatial niches possess additional transcriptomic signatures, indicating location‐specific functions [103]. For instance, cpMs adjacent to vessels express *Slc40a1/Ferroportin*, an iron exporter, suggesting their potential roles in iron metabolism [103]. These observations mark the beginning of understanding the specific transcriptomic profiles and behaviors of spatially distinct macrophages.

### 5.2 cpMs in Motion to Inflammation and Injury

Mature cpMs constantly explore their surroundings. ChP stromal macrophages and epiplexus cells differ in baseline movements [109]. ChP stromal macrophages have relatively stationary cell bodies but highly motile processes, suggesting a surveillance role [109]. These processes frequently contact blood vessels in the ChP stroma, taking up foreign material from the circulation [109]. In contrast, epiplexus cell bodies exhibit significant mobility and saltatory movements, traveling on the ChP surface at speeds of up to hundreds of micrometers per hour [109].

cpMs respond to local or peripheral stimulation, becoming activated and migrating to sites of inflammation or injury. In a peripheral inflammation model using lipopolysaccharide (LPS), ChP stromal macrophages extend along nearby vessels within 45–60 min post‐LPS delivery, potentially offering additional brain protection against harmful blood‐borne signals [109]. Conversely, epiplexus macrophages do not change morphology in response to peripheral LPS but are the major cpMs subpopulation responding to focal injury, becoming activated and migrating to injury sites [109].

However, this migration is not entirely protective. During embryonic development, abnormal activation and increased motility of cpMs can damage brain tissue structures, impairing the ChP barrier function [110]. This impairment leads to a failure to protect against harmful compounds, playing a significant role in the development of neurodevelopmental psychiatric disorders such as ASD [111, 112, 113, 114]. When researchers induced maternal immune activation at E12.5, they observed elevated levels of pro‐inflammatory cytokines, particularly chemokines C‐C motif chemokine ligand (CCL) 2, in the embryonic CSF and ChP. Macrophages within the ChP stroma responded to the increased CCL2 levels by enhancing protrusion formation and motility, leading to increased migration across the embryonic epithelial ChP BCSFB via “hotspots” at the distal tips of ChP villi. This enhanced motility disrupted the ChP barrier, characterized by loosened tight junctions. Subsequent brain tissue analysis at birth revealed disorganized cortical patches along the dorso‐lateral cerebral cortex, indicating that an excess of ChP‐derived CCL2 negatively impacts fetal brain development [110]. Collectively, cpMs exhibit diverse movement patterns and can have different effects on the ChP barrier during development and maturation.

### 5.3 Variable Roles of cpMs in Immune Surveillance

The ChP is suggested as an entry site for pathogens [115]. Located at the BCSFB interface, the transcriptomic profiles and motile behaviors of cpMs imply their active role in ChP immunity and barrier function. Cell adhesion molecules ICAM‐1 and vascular cell adhesion molecule 1 (VCAM‐1), which mediate immune‐cell migration across the BCSFB into the CNS, are exclusively located on the apical surface of ChP epithelial cells [116, 117, 118]. Additionally, ChP epithelial cells express chemokine CCL20 and the cell‐surface proteoglycan syndecan‐1, mediating the migration of CCR6^+^ IL‐17^−^ producing helper T cells (T_H_17 cells) into the CNS [119, 120]. This allows adult cpMs, possessing an antigen‐presenting signature (e.g., *Cd74*), to interact functionally with T cells during neuroinflammation [121].

cpMs have a high capacity for identifying and engulfing diverse molecules [18]. During embryonic development, cpMs are often found near apoptotic cells, indicating their role in clearing cellular debris [122]. Mature cpMs can swiftly phagocytose specific molecular weight tracers, such as Indian ink, Thorotrast, and ferritin, within minutes after intraventricular injection [109, 123]. In human fetal and childhood hydrocephalus, the number and activation of cpMs increase, with higher proportions of activation linked to worse outcomes [124, 125]. Mutations in *Csf1r*, resulting in reduced macrophages, consistently lead to hydrocephalus in *Csf1r* ^−/−^ mice, 1‐year‐old *Csf1r* ^+/−^ mice and humans with biallelic *CSF1R* mutations, indicating a protective role of cpMs in fetal and childhood hydrocephalus [126, 127, 128].

However, in adult secondary hydrocephalus, cpMs primarily promote disease progression through interactions with ChP epithelial cells [129]. Bacteria expressing LPS, which are common pathogens in post‐infectious hydrocephalus (PIH), and intraventricular hemorrhage‐derived hemoglobin, a common cause of post‐hemorrhagic hydrocephalus (PHH), stimulate cpMs via toll‐like receptor 4 (TLR4). As first responders, cpMs accumulate and proliferate at the apical ChP surface and stromal compartment, accompanied by the recruitment of peripheral blood monocytes. These macrophages exhibit amoeboid shapes, increased CD68/ED1 positivity, and robust innate immune responses, elevating cytokine levels in the CSF. These cytokines stimulate their receptors on ChP epithelial cells, leading to the activation of multiple ion and water transport proteins, resulting in increased CSF secretion and ventriculomegaly [129].

In conclusion, cpMs act as first responders in host defense and CNS pathology. Their actions can be beneficial or detrimental depending on the disease context. Further research is needed to fully understand their roles and therapeutic potential.

## 6 Prospects for Future Research of BAMs

Currently, the embryogenesis and heterogeneity of BAMs have not been fully elucidated. Future research on BAMs should focus on several critical areas. First, it is essential to understand their precise origins and how different subpopulations are influenced by their specific microenvironments during development. Additionally, exploring the mechanisms behind the maintenance and replenishment of BAM subgroups will shed light on their sustainability and functionality. Furthermore, it is crucial to investigate whether there is intragroup heterogeneity within subpopulations due to different anatomical locations, which may result in varied gene expression and functions. Other factors, such as systemic exposure to immune signals during homeostasis, external and intrinsic microbiomes, and gender, may also affect BAMs and shape their heterogeneity in a steady state, which requires further investigation.

The interactions between BAMs and other cells remain unclear. Specifically, how BAMs collaborate with both CNS‐resident immune cells and peripheral immune cells requires further clarification. Moreover, understanding whether BAMs interact with stromal cells to perform their functions and how their activity can be precisely regulated is equally important. Additionally, different BAM subtypes may potentially play roles in the same disease simultaneously, but whether their functions are antagonistic or synergistic, and whether they interact with each other, remains to be explored. Gaining this knowledge could provide critical insights into maintaining local homeostasis and ensuring effective immune responses within the CNS.

Recently, comprehensive single‐cell RNA analysis of immune cells in AD identified a new subset of microglia, known as disease‐associated microglia (DAM), which display a unique transcriptional and functional signature [108]. DAM are associated with the expression of specific genes linked to AD and other neurodegenerative conditions [108, 130, 131]. For instance, TREM2, a receptor required for DAM activation, is essential for DAM to detect and respond to neurodegenerative cues [132, 133]. DAM exhibit a specialized sensory mechanism to detect neural tissue damage in the form of neurodegeneration‐associated molecular patterns, playing crucial roles in AD pathologies [108]. Do BAMs undergo similar transcriptional and functional changes in different diseases? Is there a subset of “disease‐associated BAMs” that are present only in specific diseases, expressing disease‐specific genes and performing distinct functions during disease progression? Further investigation is urgently needed to clarify the existence of “disease‐associated BAMs,” their sensory mechanisms, signaling pathways, regulatory checkpoints, and their involvement in various brain diseases.

Clinically, BAMs could offer promising targets for therapies aimed at reducing harmful inflammation while promoting beneficial immune responses. They could serve as biomarkers for early detection and monitoring of CNS diseases, providing non‐invasive methods to track disease progression. Additionally, modulating the activity of BAMs in early disease stages, or before the disease occurs at all, may present a promising strategy to prevent the accumulation of the CNS damage. Furthermore, harnessing the regenerative potential of BAMs could enhance recovery in patients with CNS injuries or neurodegenerative diseases. By advancing our understanding of BAMs' specific roles and mechanisms, we can develop targeted therapies for a range of neuroinflammatory and neurodegenerative conditions.

## 7 Conclusion

In recent years, the study of BAMs has become central to understanding the immune environment of the CNS. Advances in single‐cell technologies and transcriptomic analyses have highlighted the heterogeneity and functional diversity of BAMs, demonstrating their unique contributions across different CNS compartments. Research now shows that BAMs are active participants in the brain's immune defense, involved in processes such as waste clearance, immunological surveillance, and regulation of vascular permeability. Their roles in neurological diseases suggest potential therapeutic implications for modulating BAM activity.

Despite these advancements, challenges remain. Key questions include delineating the precise functions of BAMs in health and disease, understanding their interactions with other CNS cells, and effectively harnessing their potential for therapeutic interventions. Continued research is essential to fully uncover their roles and leverage their potential, promising new avenues for diagnosing, treating, and preventing neurological diseases.

## Conflicts of Interest

The authors declare no conflicts of interest.

## Data Availability

The authors have nothing to report.
